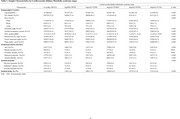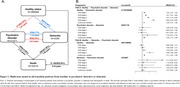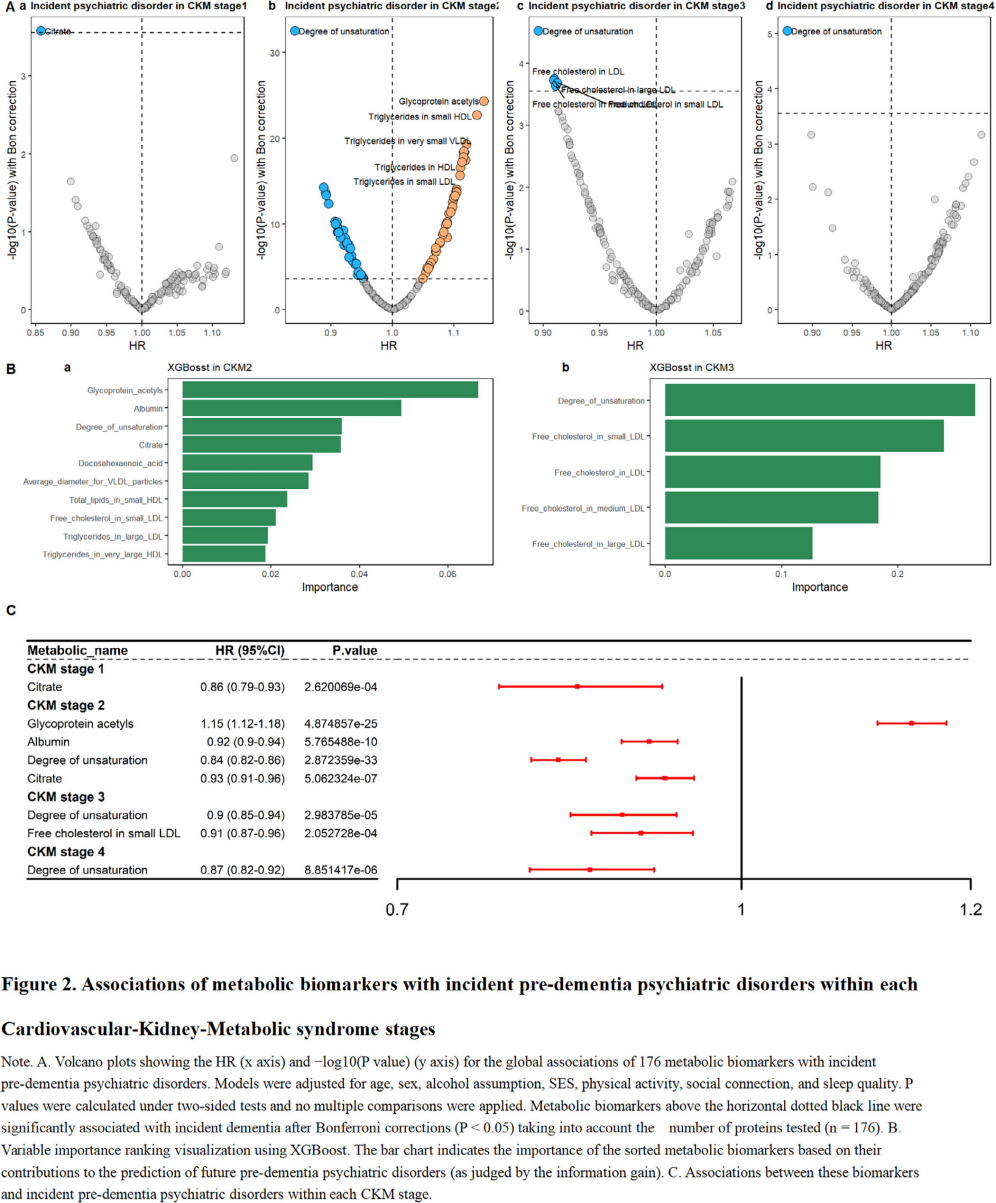# Associations of Cardiovascular‐Kidney‐Metabolic Syndrome with Psychiatric Disorders and Dementia in Midlife and Older adults in the UK Biobank

**DOI:** 10.1002/alz70860_097185

**Published:** 2025-12-23

**Authors:** Xuhao Zhao, Zhiying Lin, Haoran Zhang, Christopher Chen, Hongwei Ji, Zuyun Liu, Shaohua Hu, Xin Xu

**Affiliations:** ^1^ School of Public Health, the Second Affiliated Hospital of School of Medicine, Zhejiang University, Hangzhou, Zhejiang, China; ^2^ School of Public Health, The Second Affiliated Hospital of School of Medicine, Hangzhou, Zhejiang, China; ^3^ Memory Aging and Cognition Center, National University Health System, Singapore, Singapore; ^4^ Yong Loo Lin School of Medicine, National University of Singapore, Singapore, Singapore; ^5^ Beijing Visual Science and Translational Eye Research Institute (BERI), Beijing Tsinghua Changgung Hospital, Tsinghua Medicine, Beijing, Beijing, China; ^6^ Tsinghua Medicine, Tsinghua University, Beijing, Beijing, China; ^7^ School of Public Health and the Second Affiliated Hospital of School of Medicine, Zhejiang University, Hangzhou, Zhejiang, China; ^8^ Department of Psychiatry, the First Affiliated Hospital, Zhejiang University School of Medicine, Hangzhou, Zhejiang, China; ^9^ Nanhu Brain‐computer Interface institute, Hangzhou, Zhejiang, China

## Abstract

**Background:**

Cardiovascular‐Kidney‐Metabolic (CKM) syndrome describes pathological interactions among metabolic risk factors, chronic kidney disease, and cardiovascular dysfunction. These conditions are shared risk factors for psychiatric disorders and dementia. This study examined the associations of CKM syndrome with psychiatric disorders and dementia in middle‐aged and older adults.

**Method:**

Using data from the UK Biobank, we included participants free of psychiatric disorders and dementia at baseline. CKM syndrome was categorized into five stages (0 to 4) based on AHA definitions. Psychiatric disorders (psychotic, bipolar, depressive, and anxiety disorders) and dementia (Alzheimer's and vascular dementia) were identified using ICD‐10 codes. Multi‐state models analyzed the impact of CKM on transitions from healthy status to psychiatric disorders and dementia. Competing risk (death) models assessed the associations of CKM with specific psychiatric disorders and dementia. Additionally, Cox regression models and XGBoost classifiers were employed to identify key metabolomics associated with CKM stage‐related outcomes.

**Result:**

Among 389,314 participants, CKM stages were distributed as follows: stage 0 (10.0%), stage 1 (8.0%), stage 2 (57.6%), stage 3 (17.9%), and stage 4 (6.5%). Multi‐state model results indicated that each one‐stage increment in CKM stage was associated with higher hazards of psychiatric disorders (Healthy → Psychiatric Disorder: HR=1.26, 95% CI: [1.24, 1.29]) and subsequent transition to dementia (Psychiatric Disorder → Dementia: HR=1.30, 95% CI: [1.41, 1.49]). However, each CKM stage increment increased the hazards of directly developing dementia (Healthy → Dementia: HR=1.26, 95% CI: [1.31, 1.49]) but was not linked to subsequent psychiatric disorders. Competing risk analyses revealed that worsening CKM stages were associated with greater hazards of developing pre‐dementia psychiatric disorders, including bipolar disorder, depressive disorder, and anxiety disorder whilst only advanced CKM stages (CKM stage 3/4) were associated with all‐cause, Alzheimer's and vascular dementia. We identified several key predictors of pre‐dementia psychiatric disorders at different CKM stages (e.g., citrate at CKM stages 1 and 2; degree of unsaturation at CKM stages 3 and 4).

**Conclusion:**

CKM syndrome is associated with pre‐dementia psychiatric disorders and dementia, emphasizing the need for regular monitoring and early intervention to manage CKM progression and reduce geriatric neuropsychiatric disturbances.